# The relationships between perceived ICT convenience patterns, attitudes toward aging, and multidimensional health outcomes among older adults

**DOI:** 10.3389/fpubh.2026.1955930

**Published:** 2026-09-09

**Authors:** Zhibin Li, Ziheng Song

**Affiliations:** 1 School of Public Policy and Administration, Xi’an Jiaotong University, Xi’an, China; 2 Columbia Uplift, Lisle, IL, United States; 3 Dartmouth College, Hanover, NH, United States

**Keywords:** attitudes toward aging, digital divide, latent class analysis, multidimensional health, perceived convenience of ICT

## Abstract

**Background/objectives:**

In the digital era, research on the digital divide has largely focused on inequalities in access and skills, with less attention paid to differences in the benefits older adults derive from information and communication technology (ICT), the third-level digital divide. Nevertheless, how these heterogeneous patterns relate to health outcomes remains unclear.

**Methods:**

Based on the 2023 wave of the China Longitudinal Aging Social Survey (CLASS), this study employed person-centered latent class analysis (LCA) to identify potential classes of perceived convenience of ICT (PCICT) and further examined factors associated with these classes and these classes’ associations with older adults’ health, and explored the mediating role of attitudes toward aging (ATA).

**Results:**

Five latent classes of PCICT were identified: All-domains Beneficiaries (21.73%), Foundational-domains Beneficiaries (19.02%), Extended-domains Frustrated (12.76%), Compound Experience (31.71%), and Unaffected (14.77%). The distribution of these classes varied according to individual, family, and community characteristics. The All-domains Beneficiaries and Foundational-domains Beneficiaries classes were generally associated with more favorable health outcomes, whereas the Extended-domains Frustrated class was associated with more depressive symptoms. Furthermore, significant indirect associations through ATA were observed between PCICT classes and health outcomes.

**Conclusion:**

This study reveals the heterogeneity of the third-level digital divide among older adults by identifying different classes of PCICT. These classes were differentially associated with multidimensional health outcomes. ATA partly accounted for these associations. Our evidence underscores the importance of paying attention to different benefits of ICT and fostering positive ATA among older adults.

## 1 Introduction

Population aging has emerged as a major health and socioeconomic concern worldwide. China has the largest older-adult population and also ranks among the fastest-aging nations globally (1). At the same time, information and communication technology (ICT) is significantly reshaping society and becoming increasingly embedded in daily life. As “digital immigrants,” older adults’ interactions with ICT are complex. Digitalization can serve as a resource for successful aging or a source of stress and exclusion. On the one hand, ICT brings opportunities (such as accessing information and maintaining social connections) (2, 3); on the other hand, it also presents challenges related to digital inequality. Although prior studies have mainly focused on ICT access and skills (4), these factors do not necessarily ensure that older adults can obtain meaningful benefits from ICT (5). As ICT further penetrates into older adults’ daily lives, it is time to focus on their perceived convenience of ICT (PCICT), which refers to older adults’ subjective evaluation of the extent to which ICT makes different domains of everyday life more convenient. This issue is particularly important in China, where mobile payments and the digitalization of essential public services have made ICT increasingly embedded in everyday life. In such a high-penetration environment, the gap between ICT use and meaningful benefit acquisition may be closely linked to older adults’ well-being.

Previous studies have explored the “third digital divide” (4, 5). However, several research gaps remain. First, existing research has documented differences in the outcomes and benefits of ICT (5–8). However, little is known about whether older adults exhibit distinct patterns of PCICT across different domains of daily life. Second, previous studies have mainly relied on small-sample qualitative research or variable-centered approaches (9–11). Although these studies have provided important insights into older adults’ ICT experiences, such approaches are limited in identifying heterogeneous and person-centered patterns of PCICT. Third, few studies have empirically examined the associations between different patterns of PCICT and multidimensional health outcomes. Moreover, little is known about whether attitudes toward aging (ATA), an important psychosocial factor associated with health in later life, may partly account for these associations.

To address these gaps, this study first employs latent class analysis (LCA) to identify distinct PCICT classes. Second, this study examines factors associated with class membership and the associations between these classes and multidimensional health outcomes. Finally, this study further examines the indirect associations between PCICT classes and health outcomes through ATA. Specifically, this study addresses the following four research questions:

(1) What latent classes of PCICT can be identified?
(2) What factors are associated with different PCICT classes?
(3) How are different PCICT classes associated with multidimensional health outcomes?
(4) Are PCICT classes indirectly associated with multidimensional health outcomes through ATA?

## 2 Literature review

### 2.1 The evolution of the digital divide: from access to benefit

The digital divide theory focuses on analyzing the disparities in access to and use of ICT across different groups, and these disparities are jointly influenced by socioeconomic variables including income, educational attainment, and geographic location (12). As a key term of digital society, “Digital Divide” has moved from physical access disparities (first digital divide) to skill and usage gaps (second digital divide), and ultimately to inequalities in the benefits (third digital divide) (4). Initially, the concept of “digital divide” was used to describe the disparities between individuals who used the computer or internet and individuals who could not access the computer or internet (13), which was often measured as a dichotomous variable (internet use and nonuse). With the development of ICT, more individuals are accessing ICT, exhibiting considerable heterogeneity among Internet users (14). Research has increasingly focused on disparities in digital skills and usage patterns, known as the second-level digital divide (4, 15, 16).

Taken together, research into the first and second digital divide has increased significantly over the past 20 years (16–18). However, there is a growing recognition that inequalities also exist in the outcomes of internet access or internet skills, which constitutes the third digital divide at the effect level (5, 19). On the one hand, some scholars emphasize the need to analyze disparities in the objective consequences resulting from differences in internet access or usage (4, 9). For example, a study on Chinese older adults found that the impact of online activities on psychological well-being varied based on objective factors, such as employment status and residence (9). On the other hand, others emphasize subjective utility differences (utility gap) in internet use. They argue that understanding an individual’s perceived convenience is critical, because it shows digital inequality in their life experiences (6, 7). For example, a study on the Dutch population examined the types of internet engagement that lead to different economic, cultural, social, and personal outcomes (19). Despite these contributions, a comprehensive understanding of how older adults subjectively perceive the convenience of ICT across various life domains is still lacking.

ICT has become an integral part of modern life; therefore, it is time to consider exactly who benefits from ICT and in what aspects. Against this growing interest in the third-level digital divide, this study examines the third digital divide by focusing on PCICT. PCICT is more concrete than the general perceived importance (20). It captures the gap between what ICT could offer and what people actually gain in daily life. In other words, it reflects how people value ICT’s convenience across different life domains (21, 22). PCICT is conceptually similar to well-known psychological constructs, for example, perceived ease of use from the Technology Acceptance Model (23). However, PCICT is not equivalent to perceived ease of use. Perceived ease of use mainly concerns whether a specific technology is easy to operate. In contrast, PCICT focuses on whether older adults perceive ICT as making different domains of everyday life more convenient. By shifting attention from the usability of a specific technology to the convenience and meaningful benefits that older adults perceive across different life domains, PCICT provides a benefit-oriented perspective that corresponds closely to the third digital divide’s concern with inequalities in the outcomes and benefits derived from ICT use. Specifically, in this study, based on the evaluation of eight main life domains where ICT was applied, we employed the LCA approach to explore the distinct latent classes of PCICT.

### 2.2 Heterogeneity in older adults’ ICT experiences

For older adults, ICT is a double-edged sword in daily life. On one hand, numerous studies have shown that it can enhance their well-being. A systematic review highlighted that ICT caters to the medical care, spiritual comfort, and entertainment needs of older adults (24) and can facilitate everyday life, social connectedness, aging at home, well-being, and dignified care (25). However, technology is not uniformly beneficial for older adults (26). Research has found that older adults may experience difficulties, frustration, and anxiety related to ICT (27). Some studies have reported that mobile technologies can both enhance well-being and pose problems for older adults (28). This dual nature of ICT experience is powerfully illustrated in qualitative research. For instance, a study using interpretative phenomenological analysis found that the lived experience of older adults centered on two superordinate themes: digital technology as a tool for both empowerment and disempowerment (29). Similarly, another study concluded that older adults cannot be viewed as a homogeneous group in terms of their experience with ICT. Specifically, some older adults felt frustrated or frightened by ICT, while others felt comfortable and proud about using it (30).

Taken together, this body of work effectively demonstrates that the ICT experiences of older adults are highly diverse. However, these studies, especially qualitative ones, excel at describing the existence and richness of this diversity; they are not designed to systematically identify distinct, empirically derived patterns of experience or to estimate their prevalence within a larger population. We do not yet have a clear typology of what these distinct patterns look like. Therefore, it is necessary to use the LCA approach to develop an evidence-based typology of older adults’ PCICT by identifying unobserved subgroups of individuals who share typical perception patterns.

### 2.3 Factors associated with the digital divide among older adults

The factors associated with the digital divide have long been a topic of debate. In the early stages of internet and digital technology development, researchers widely believed that technological barriers were the primary explanation for the digital divide (31). With the rapid penetration of mobile ICT, the digital divide cannot be explained solely by technological factors but is also associated with social factors, such as socio-demographic characteristics (4, 17, 32). Among these characteristics, age is seen as a key factor associated with the digital divide; thus, the age-related digital divide among older adults has gained public concern (33, 34). Nonetheless, in recent decades, the age-related digital divide has been narrowing (35).

Moreover, further studies have shown that the digital divide is closely linked to other factors such as education, income levels, attitudes toward ICT, Big Five personality traits, social support, type of technology, digital training, rights, infrastructure, and more (17, 33, 36, 37). Additionally, social networks and resources seem to have a limited association with the second-level digital inclusion of older users (38). However, these studies mainly focus on the first and second digital divide (17, 33, 38). Currently, there is a lack of empirical research that comprehensively considers the various factors associated with the third digital divide.

### 2.4 The association between the digital divide and health outcomes

The digital divide is closely linked to health and well-being (4, 39). Various studies have shown that internet use and digital skills are connected with better physical health among older adults by providing access to online health information (40) and facilitating healthy lifestyles (41). They also promote and facilitate social and mental health by helping individuals stay connected with family and friends and offering resources for entertainment (42–44).

However, this relationship is not always positive. In other words, access to ICT and owning digital skills do not guarantee beneficial outcomes. The quality of the digital experience matters, and negative experiences can potentially harm the well-being of older adults (26). This points to a deeper process, especially concerning mental health. Drawing on the Stress Process Model (45), frustrating interactions with technology can serve as a chronic stressor. Older adults often struggle with ICT use and experience higher levels of technostress (46–48), which can lead to feelings of incompetence and frustration, undermining their self-efficacy and resulting in negative psychological outcomes such as anxiety and depression (49, 50). The psychological cost of a frustrating digital journey has been largely ignored in previous research.

### 2.5 The mediating role of attitudes toward aging

Within the third digital divide framework, PCICT captures heterogeneity in the benefits older adults perceive from ICT across different domains of daily life, and these differences may be associated with health outcomes. However, little is known about the psychosocial processes that may be involved in these associations. A previous study found that older adults’ engagement with digital technology was associated with how they perceived their own aging (51). Stereotype Embodiment Theory (SET) provides a useful perspective in this regard by highlighting attitudes toward aging (ATA) as an important psychosocial factor associated with health in later life.

Attitudes toward aging (ATA) refer to individuals’ perceptions and evaluations regarding the aging process and being older (52). ATA is shaped by personal experiences, social interactions, intergroup stereotypes, cultural values, and social structures (53). ATA has been recognized as an important psychological factor associated with health behaviors, psychosocial adjustment, and even physical decline in later life (54, 55). SET adopts a lifespan perspective to elucidate the distinct impact of ATA on the aging process (56). According to this theory, age-related stereotypes are internalized across the lifespan and exert their influence on health outcomes through psychological, behavioral, and physiological pathways (57–59). Specifically, negative ATA is associated with reduced self-efficacy, lower social participation, and heightened stress and emotional reactivity, indirectly accelerating biological aging (60, 61). In contrast, positive ATA increases self-efficacy and control beliefs, which in turn promote health behaviors such as physical activity and healthy eating (62). Taken together, these findings provide a theoretical basis for considering ATA as an important psychosocial factor associated with health in later life.

In the digital context, previous empirical studies have found an association between internet use and more positive ATA (63). Older Chinese internet users tend to hold more positive perceptions of their aging process (64, 65). Another study in the United States also observed that increased internet use was associated with more positive self-perceptions of aging (66). However, older adults’ digital technology experiences are heterogeneous (30), and how different digital technology experiences relate to older adults’ ATA remains underexplored in existing research. Different ICT experiences may also be differently associated with older adults’ attitudes toward aging. Positive ICT experiences may be associated with older adults’ greater sense of autonomy, social inclusion, and competence (67, 68), thereby fostering more positive ATA. In contrast, negative ICT experiences may reinforce stereotypes of older adults (48, 69) and may be associated with more negative ATA. Given that ATA is associated with both digital experiences and health outcomes, ATA may partly account for the associations between PCICT classes and health outcomes.

### 2.6 Conceptual framework

**Figure 1:**
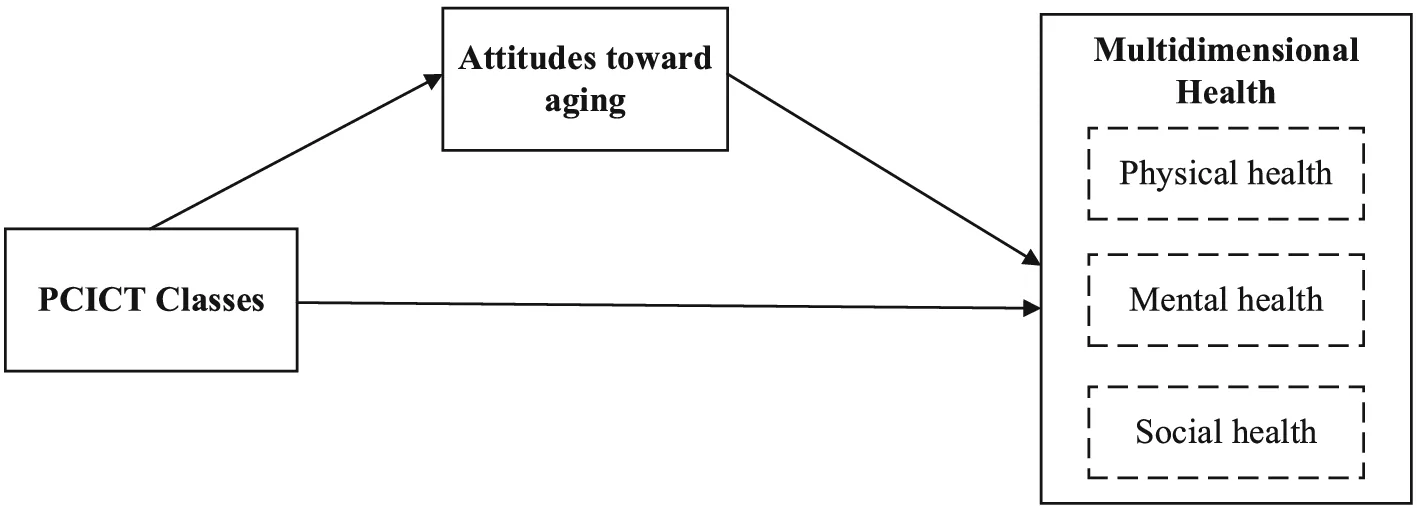
Conceptual model.

Based on the above theoretical analysis, this study integrates Digital Divide Theory and Stereotype Embodiment Theory into a conceptual framework linking PCICT classes, ATA, and multidimensional health outcomes (see Figure 1). In this conceptual framework, ATA is considered as a potential psychosocial factor through which PCICT classes may be indirectly associated with health outcomes. Digital Divide Theory provides the broader framework for understanding inequalities in the benefits older adults derive from ICT, including heterogeneity in PCICT, factors associated with these differences, and their associations with health outcomes. Stereotype Embodiment Theory provides a theoretical basis for considering ATA as a psychosocial factor that may partly account for the associations between PCICT classes and health outcomes. First, the framework focuses on heterogeneity in PCICT classes. Second, it examines the factors associated with class membership. Third, it examines the associations between different PCICT classes and multidimensional health outcomes. Finally, it examines whether PCICT classes are indirectly associated with health outcomes through ATA.

## 3 Materials and methods

### 3.1 Participants

**Figure 2:**
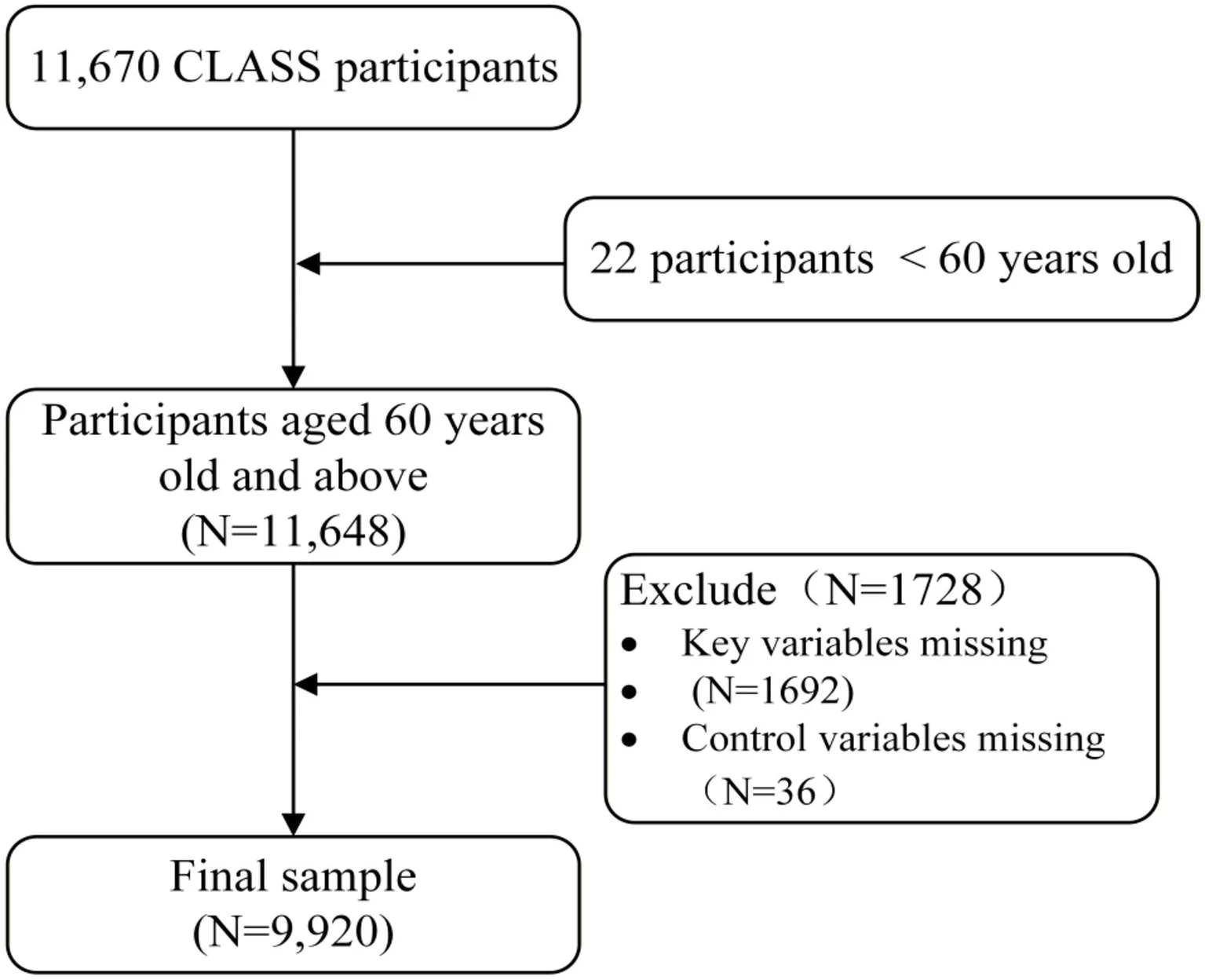
Sample selection criteria and procedure in this study.

The data used in this study were drawn from the 2023 wave of the China Longitudinal Aging Social Survey (CLASS). CLASS is a nationwide, ongoing, large-scale social survey project. CLASS selected individuals aged 60 years or older through multi-phase probability sampling. The CLASS survey regularly and systematically collects information on older adults in China, including their social, economic, health, family, and community characteristics. These data provide important empirical evidence for understanding and addressing population aging in China. More detailed information about CLASS can be found on this website (http://jkzgyjy.ruc.edu.cn/sjzy/CLASSsjsq/ade1ba3977354553a798c8f10221c2ad.htm). Of the 11,670 participants in our 2023 national survey, we excluded 22 who were under 60 and 1,728 with missing values, yielding a final sample of 9,920. The detailed information is shown in Figure 2.

### 3.2 Measures

#### 3.2.1 Perceived convenience of information and communication technology

Perceived convenience of information and communication technology (PCICT) was assessed using eight items from the CLASS 2023 questionnaire. PCICT was not treated as a pre-existing standardized scale; rather, it was operationalized as a study-specific operational measure to capture older adults’ subjective evaluations of the convenience brought by ICT in different domains of everyday life. In the CLASS 2023 questionnaire, these eight items covered two types of ICT-related life domains: foundational domains and extended domains. The foundational domains referred to relatively common everyday activities, including “social interaction,” “shopping and consumption,” “accessing news and information,” and “leisure and entertainment.” The extended domains referred to more specialized or service-oriented activities, including “travel and tourism,” “health services,” “investment and financial management,” and “learning and training.” These items directly assessed perceived ICT-related convenience; for each domain, respondents selected one of the following four response options regarding the perceived convenience of ICT: “Convenience,” “Inconvenience,” “No impact,” and “Uncertain.” These four response options were all retained as original categorical responses in the LCA model, rather than being recoded into binary indicators, in order to preserve the distinction between perceived convenience, perceived inconvenience, perceived no impact, and uncertainty. These eight categorical items served as the manifest indicators for the LCA model.

#### 3.2.2 Multidimensional health

Following the World Health Organization’s multidimensional definition of health, this study assessed older adults’ health across three corresponding dimensions: physical, mental, and social health (70). This framework is also relevant to the study of ICT among older adults, as ICT is increasingly connected with access to health information and services, psychological well-being, and opportunities for social connection and participation (71, 72). Previous studies examining Internet use and multidimensional health have similarly considered these three dimensions (8, 73).

Following previous studies (8, 74), physical health was evaluated through self-rated health, a comprehensive evaluation of the physical health of older adults (75, 76). Compared with more specific indicators such as ADL limitations and chronic conditions, self-rated health is a broader subjective assessment of health status (76) and may be responsive to ICT-related experiences (8). Specifically, self-rated health was assessed using the question “How do you feel about your current physical health?”, with response options ranging from 1 (“very unhealthy”) to 5 (“very healthy”).

Mental health was assessed by depressive symptoms and life satisfaction. Depressive symptoms were assessed using a revised version of the Center for Epidemiologic Studies Depression Scale (CES-D) (77, 78). This scale included nine items, such as “feelings of loneliness,” “sadness,” “feelings of worthlessness,” and others. Each item was rated on a 3-point scale (1 = Never, 2 = Sometimes, 3 = Often). We reverse-coded three positive items so that all items pointed the same way. Total scores thus ranged from 9 to 27. Higher scores indicate more severe depressive symptoms, and the scale’s Cronbach’s α was 0.817. Life satisfaction was assessed by a single-item measure, “In general, are you satisfied with your current life?” The options were “1= very dissatisfied,” “2 = dissatisfied,” “3 = fair,” “4 = satisfied,” and “5 = very satisfied.” Prior research has validated the same measurement approach among older adults (79, 80).

Social health was measured using the Social Adaptation Scale (81). Previous studies have validated this scale and used it across many domains (74, 82). The scale includes four positive items and four negative items. Each item has five options ranging from “1 = Strongly disagree” to “5 = Strongly agree”. Negative items are scored in reverse and combined to create the total score (range: 8–40; higher scores indicate better social health). The scale’s Cronbach’s α was 0.750.

#### 3.2.3 Attitudes toward aging

Attitudes toward aging (ATA) were measured using a seven-item ATA scale (83). Each item was scored from 1 (totally disagree) to 5 (totally agree). Four negative items were reverse-coded. The seven items were summed to give a total score of 7–35, with higher scores representing more positive attitudes toward aging. In this study, the Cronbach’s alpha for the scale was 0.668.

#### 3.2.4 Correlates of PCICT class

Factors associated with PCICT class were examined at the individual, family, and community levels. To be more specific, individual-level factors mainly related to the basic demographic characteristics of older adults, including age (1 = 60–69 years, 2 = 70–79 years, 3 = 80 years and above), gender (0 = female, 1 = male), education level (1 = illiterate, 2 = primary education or below, 3 = junior high school or above), marital status (1 = have a spouse, 2 = other), chronic condition (1 = at least one chronic disease, 0 = no disease) and annual income (ln-transformed). Activities of daily living (ADL) included dressing, bathing, eating, toileting, bed-to-chair transfer, and indoor walking. Each item had three response options: 1 (independent), 2 (needs some help), or 3 (fully dependent). The total score ranges from 6 to 18, with higher scores indicating greater ADL limitations. ADL limitations and chronic conditions were included as health-related factors associated with PCICT class membership because they capture relatively stable health constraints that may be relevant to older adults’ engagement with digital technologies and their ability to obtain convenience from ICT (84, 85). At the family level, we considered the number of children and whether participants lived alone (0) or with family members (1). At the community level, we considered rural–urban residence (rural = 0, urban = 1) and region (western = 0, central = 1, eastern = 2).

### 3.3 Analytical strategy

First, LCA was employed to uncover underlying patterns in respondents’ PCICT. LCA is a statistical method to identify latent classes using a selected set of indicators (86). Following the guidelines of LCA (87, 88), we tested a range of models, from a 1-class to a 7-class solution. Model selection considered several indicators. In this study, we used seven fit indices, including the Akaike Information Criterion (AIC), the Bayesian Information Criterion (BIC), the Sample-Size Adjusted BIC (aBIC), the Lo–Mendell–Rubin Likelihood Ratio Test (LMRT), the Bootstrapped Likelihood Ratio Test (BLRT), Relative Frequency of Smallest Class (RFSC), and entropy. Lastly, high classification quality, indicated by an entropy value of ≥ 0.80, and substantive interpretability, ensuring no class was too small (< 5%) to be generalizable, were also key considerations (88–90). After selecting the best-fitting model, the classes were interpreted and labeled based on their distinct conditional item-response probabilities. Second, we used a multinomial logit model to examine factors associated with class membership. Third, we ran ordinary least squares (OLS) models to examine the associations between PCICT classes and health outcomes. Finally, we examined the indirect associations between PCICT classes and health outcomes through ATA using the Karlson-Holm-Breen (KHB) method (91). Statistical significance was assessed using bias-corrected bootstrap confidence intervals based on 5,000 resamples. We further conducted sensitivity analyses to enhance the robustness of the analyses. Mplus 8.3 was used for the latent class analysis, whereas the remaining statistical analyses were completed in Stata version 18.0.

## 4 Results

### 4.1 Characteristics of the study population

The characteristics of the study population (N = 9,920) are presented in Table 1. Nearly half of the participants were aged 70–79 years (49%), and the sample included slightly more men (52%) than women (48%). Overall, 42% had completed junior high school or above. Most participants were married or partnered (83%), lived with family members (92%), and reported at least one chronic condition (79%). The mean log-transformed annual income was 9.51 (SD = 1.13), and the mean ADL score was 6.14 (SD = 0.83). Participants had an average of 2.08 children (SD = 1.14). Slightly more than half lived in rural areas (52%), and participants from eastern China constituted the largest regional group (40%).

**Table 1:** Descriptive statistics of the sample characteristics (N = 9,920).

Variables	Category (Range)	*N* (%)/M(SD)
Age	60–69 years old	4,027 (41%)
	70–79 years old	4,836 (49%)
	80 years old and above	1,057 (11%)
Gender	Female	4,798 (48%)
	Male	5,122 (52%)
Education level	Illiterate	1,709 (17%)
	Primary education or below	4,045 (41%)
	Junior high school or above	4,166 (42%)
Marital status	Have a spouse	8,202 (83%)
	Others	1,718 (17%)
Chronic condition	No	2,072 (21%)
	Yes	7,848 (79%)
Annual income (log-transformed)	4.61 ~ 12.89	9.51 (1.13)
Activities of daily living	6 ~ 18	6.14 (0.83)
Number of children	0 ~ 9	2.08 (1.14)
Living arrangement	Live with family	9,081 (92%)
	Live alone	839 (8%)
Residence	Rural	5,183 (52%)
	Urban	4,737 (48%)
Region	Central areas	3,374 (34%)
	Eastern areas	3,958 (40%)
	Western areas	2,588 (26%)

M, mean; SD, standard deviation.

### 4.2 Model selection of PCICT

The model fit indices are displayed in Table 2. First, across the fitted models, the AIC, BIC, and aBIC values showed a consistent downward trend as the number of classes was increased, but the decline began to level off after the five-class model. Although the six-class and seven-class models showed lower information criteria, their smallest classes accounted for only 3.4 and 1.8% of the sample, respectively. Because both proportions were below 5%, the six-class model and the seven-class model were excluded due to the potential risk of over-extraction and limited substantive interpretability (92). Second, the LMRT and BLRT results were statistically significant for models with two to five classes, indicating that the K-class model fit the data significantly better than the K-1 class model (89). Therefore, the five-class model was considered better than the four-class model. Third, based on the entropy value, the five-class model had a higher entropy than the four-class model, which means the five-class model provided more meaningful classifications of PCICT. Considering the information criteria, LMRT and BLRT results, entropy value, class size, and interpretability, the five-class model was chosen as the optimal solution.

**Table 2:** Comparison of LCA model fit indices.

Model	AIC	BIC	aBIC	LMRT	BLRT	RFSC	Entropy
1-Class	229520.41	229697.17	229617.00	–	–	–	1.000
2-Class	197776.26	198137.13	197973.46	0.000	0.000	41.2%	0.909
3-Class	184600.58	185145.57	184898.39	0.000	0.000	17.5%	0.928
4-Class	178865.33	179594.44	179263.76	0.000	0.000	14.7%	0.906
5-Class	173629.79	174543.02	174128.83	0.000	0.000	12.7%	0.916
6-Class	171250.16	172347.52	171849.81	0.000	0.000	3.4%	0.926
7-Class	170172.44	171453.91	170872.70	0.000	0.000	1.8%	0.929

AIC, Akaike Information Criterion; BIC, Bayesian Information Criterion; aBIC, Sample-Size Adjusted BIC; LMRT, Lo–Mendell–Rubin Likelihood Ratio Test; BLRT, Bootstrapped Likelihood Ratio Test; RFSC, Relative Frequency of Smallest Class; −, not applicable.

### 4.3 Latent class identification of PCICT

**Figure 3:**
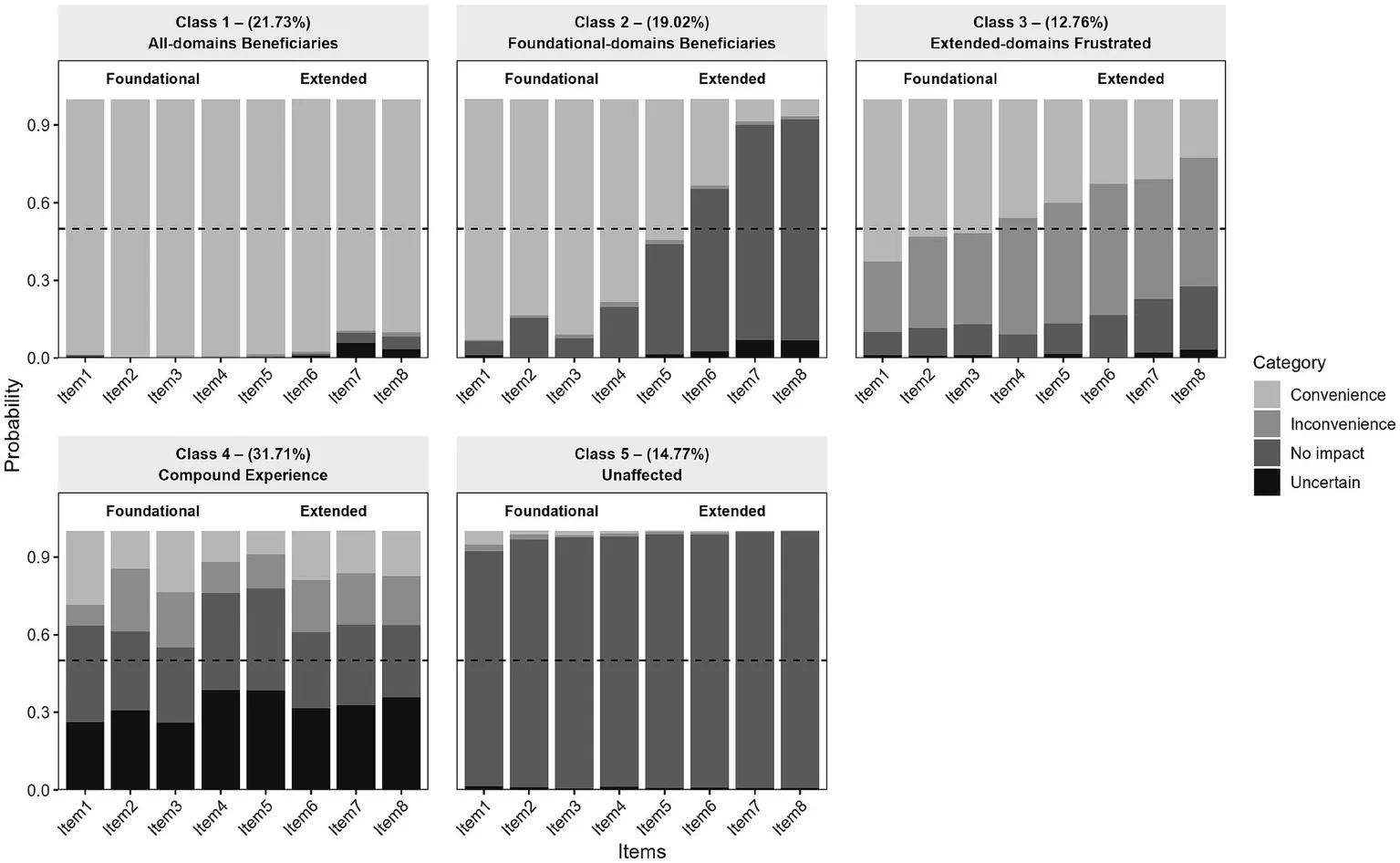
Latent class item-response probabilities for five latent classes. Items correspond to the following life domains: Item1, Social interaction; Item2, Shopping and consumption; Item3, Accessing news and information; Item4, Leisure and entertainment; Item5, Travel and tourism; Item6, Health services; Item7, Investment and financial management; Item8, Learning and training.

The estimated class prevalences and item-response probabilities for the five latent classes across the eight life domains are presented in Figure 3. The five classes were labeled according to their conditional item-response probabilities across the eight life domains. In particular, the labels reflected which response category, “Convenience,” “Inconvenience,” “No impact,” or “Uncertain,” was predominant and whether the response patterns differed between the foundational and extended domains.

The first class comprised 21.73% of the sample. Participants in this group had high probabilities of perceiving ICT as convenient across all eight life domains, covering both foundational and extended domains. We therefore named this class “All-domains Beneficiaries.”

The second class accounted for 19.02% of the sample and showed a clear distinction between the foundational and extended domains. The probabilities of reporting “Convenience” exceeded 50% in all four foundational domains. In contrast, in the four extended domains, the probabilities of reporting “No impact” were generally higher than those of reporting “Convenience.” Because perceived convenience was concentrated primarily in the foundational domains, this class was labeled “Foundational-domains Beneficiaries.”

The third class accounted for 12.76% of the sample and also showed contrasting response patterns between foundational and extended domains. This class had relatively high probabilities of reporting “Convenience” in the foundational domains but also higher probabilities of reporting “Inconvenience” in several extended domains. Because the pattern combined perceived convenience in foundational domains with perceived inconvenience in extended domains, this class was labeled “Extended-domains Frustrated.”

The fourth class was the largest, comprising 31.71% of the sample. Across the eight life domains, the probabilities were spread across “Convenience,” “Inconvenience,” “No impact,” and “Uncertain.” Because none of the four response categories was dominant, we labeled this mixed pattern of perceived ICT experiences “Compound Experience.”

The fifth class accounted for 14.77% of the sample. It was characterized by consistently high probabilities of reporting “No impact” across all eight life domains, reaching approximately 90% in all domains. Because ICT was generally perceived as having “no impact” across both the foundational and extended domains, this class was labeled “Unaffected.”

### 4.4 Multinomial logistic regression of five PCICT classes on the demographic covariates

To examine how demographic covariates were associated with PCICT classes, we first conducted Pearson’s chi-square tests and analysis of variance to describe the demographic distribution across the five classes. The results are shown in Supplementary Table S1. Second, we performed a multinomial logistic regression (93) to explore the associations of individual, family, and community factors with the five latent classes, using the “Unaffected” class as the reference group.

Table 3 reports the associations between the related factors and latent class membership. Age was associated with class membership. Compared with the “Unaffected” class, participants aged 70–79 years and those aged 80 years or older were less likely to belong to the “All-domains Beneficiaries,” “Foundational-domains Beneficiaries,” and “Extended-domains Frustrated” classes (all *p* < 0.001). Furthermore, those aged 80 and older were also significantly less likely to belong to the “Compound Experience” class (*p* < 0.001). Gender was not significantly associated with any of the four classes. Regarding education level, compared to illiterate participants, individuals with higher education levels (primary education or below; junior high school or above) were significantly more likely to belong to all four classes (all *p* < 0.001). Compared to individuals with “Other” marital status, those with a spouse were more likely to be in the “All-domains Beneficiaries” (*p* < 0.01), “Foundational-domains Beneficiaries” (*p* < 0.001), and “Extended-domains Frustrated” (*p* < 0.001) classes. Having a chronic condition was associated with a lower likelihood of being in the “All-domains Beneficiaries” (*p* < 0.001) and “Compound Experience” (*p* < 0.001) classes, but a higher likelihood of being in the “Foundational-domains Beneficiaries” class (*p* < 0.01). Higher annual income was significantly associated with a greater likelihood of belonging to all four classes (all *p* < 0.001). Higher ADL limitation scores were associated only with a greater likelihood of being in the “Compound Experience” class (*p* < 0.05).

**Table 3:** Associations of related factors with latent class membership relative to the “Unaffected” class.

Variables	All-domains Beneficiaries	Foundational-domains Beneficiaries	Extended-domains Frustrated	Compound Experience
Individual-level factors				
Age (Ref. 60–69 years old)				
70–79 years old	0.469^***^	0.454^***^	0.482^***^	0.901
	(0.396–0.556)	(0.383–0.540)	(0.401–0.579)	(0.769–1.056)
80 years old and above	0.388^***^	0.231^***^	0.332^***^	0.371^***^
	(0.297–0.507)	(0.174–0.307)	(0.248–0.443)	(0.293–0.468)
Gender (Ref. Female)	1.036	1.059	1.024	0.901
Male	(0.894–1.200)	(0.913–1.229)	(0.873–1.201)	(0.790–1.028)
Education level (Ref. Illiterate)				
Primary education or below	1.552^***^	1.651^***^	1.609^***^	1.334^***^
	(1.266–1.903)	(1.346–2.026)	(1.300–1.991)	(1.136–1.567)
Junior high school or above	3.520^***^	2.730^***^	2.689^***^	2.607^***^
	(2.776–4.464)	(2.145–3.474)	(2.084–3.469)	(2.125–3.199)
Marital status (Ref. Other)	1.449^**^	1.782^***^	1.580^***^	0.997
Have a spouse	(1.133–1.855)	(1.374–2.312)	(1.206–2.070)	(0.820–1.213)
Chronic condition (Ref. No)	0.589^***^	1.410^**^	0.958	0.584^***^
Yes	(0.487–0.712)	(1.143–1.739)	(0.773–1.188)	(0.490–0.697)
Annual income (Ln)	1.644^***^	1.769^***^	1.575^***^	1.467^***^
	(1.534–1.763)	(1.649–1.898)	(1.462–1.696)	(1.383–1.557)
Activities of daily living	0.979	1.078	0.917	1.070^*^
	(0.886–1.082)	(0.990–1.174)	(0.809–1.038)	(1.003–1.142)
Family-level factors				
Number of children	0.861^***^	1.006	1.019	1.052
	(0.800–0.926)	(0.936–1.082)	(0.944–1.099)	(0.991–1.118)
Living arrangement (Ref. Live alone)	1.100	1.599^**^	1.583^**^	1.290
Live with family	(0.791–1.529)	(1.142–2.238)	(1.124–2.229)	(1.000–1.664)
Community-level factors				
Residence (Ref. Rural)	1.508^***^	1.332^***^	1.100	0.917
Urban	(1.273–1.786)	(1.124–1.579)	(0.916–1.320)	(0.788–1.067)
Region (Ref. Western areas)				
Central areas	0.466^***^	1.373^**^	0.796^*^	0.837^*^
	(0.385–0.563)	(1.127–1.673)	(0.654–0.969)	(0.715–0.980)
Eastern areas	0.794^*^	1.572^***^	0.776^*^	0.608^***^
	(0.652–0.967)	(1.271–1.944)	(0.624–0.965)	(0.507–0.728)
Constant	0.001^***^	0.000^***^	0.005^***^	0.108^***^
	(0.000–0.003)	(0.000–0.001)	(0.002–0.017)	(0.047–0.248)
Observations	9,920	9,920	9,920	9,920
Pseudo *R*^2^	0.0897	0.0897	0.0897	0.0897

Coefficients reported in this table are the relative risk ratios (RRRs). Ref. = Reference Category. () = 95% confidence interval. *p < 0.05, **p < 0.01, ***p < 0.001.

Regarding family factors, having more children was associated with a lower likelihood of belonging to the “All-domains Beneficiaries” class (*p* < 0.001) but was not significantly associated with membership in the other classes. Participants living with family were more likely than those living alone to belong to the “Foundational-domains Beneficiaries” and “Extended-domains Frustrated” classes (both *p* < 0.01). Additionally, significant urban–rural and regional differences were observed. Urban residents were more likely than rural residents to belong to the “All-domains Beneficiaries” and “Foundational-domains Beneficiaries” classes (both *p* < 0.001). Compared to residents in the western areas, those in central and eastern regions were less likely to be in the “All-domains Beneficiaries” class but more likely to be in the “Foundational-domains Beneficiaries” class. Furthermore, residents from central (*p* < 0.05) and eastern (*p* < 0.001) regions were also less likely to belong to the “Compound Experience” class.

### 4.5 PCICT class differences in distal health outcomes

Table 4 displays the associations between the PCICT classes and health outcomes. Compared with the “Unaffected” class, the “All-domains Beneficiaries” and “Foundational-domains Beneficiaries” classes were generally associated with more favorable health outcomes. Members of the “All-domains Beneficiaries” class reported better self-rated health (*β* = 0.246, *p* < 0.001), fewer depressive symptoms (*β* = −1.185, *p* < 0.001), higher life satisfaction (*β* = 0.270, *p* < 0.001), and better social adaptation (*β* = 2.354, *p* < 0.001). The “Extended-domains Frustrated” and “Compound Experience” classes were also associated with higher life satisfaction and better social adaptation than the “Unaffected” class. Notably, the “Extended-domains Frustrated” class was not related to physical health but was associated with a higher level of depressive symptoms (*β* = 0.867, *p* < 0.001) relative to the “Unaffected” class. Moreover, the “Compound Experience” class showed no association with physical health or depressive symptoms when compared to the “Unaffected” class.

**Table 4:** Associations of class types and health outcomes.

Variables	SRH	DS	LS	SA
PCICT (Ref. Unaffected)				
All-domains beneficiaries	0.246^***^	−1.185^***^	0.270^***^	2.354^***^
	(0.026)	(0.112)	(0.026)	(0.139)
Foundational-domains Beneficiaries	0.152^***^	−0.592^***^	0.120^***^	0.586^***^
	(0.026)	(0.113)	(0.026)	(0.141)
Extended-domains frustrated	0.004	0.867^***^	0.114^***^	0.724^***^
	(0.028)	(0.121)	(0.028)	(0.151)
Compound experience	0.032	−0.068	0.092^***^	1.020^***^
	(0.023)	(0.100)	(0.023)	(0.124)
Control variables	YES	YES	YES	YES
Constant	3.860^***^	15.903^***^	3.428^***^	20.197^***^
	(0.102)	(0.439)	(0.100)	(0.547)
Observations	9,920	9,920	9,920	9,920
*R* ^2^	0.180	0.185	0.096	0.125
F	114.8	118.6	55.17	74.48

Standard errors in parentheses, ***p < 0.001, **p < 0.01, *p < 0.05, SRH, Self-rated health, DS, Depressive symptoms, LS, Life satisfaction, SA, Social adaptation.

### 4.6 Mediation analysis of ATA

To investigate whether ATA partly accounted for the associations between PCICT classes and health outcomes, we employed the KHB method. The statistical significance of the indirect effects was assessed using bias-corrected bootstrap confidence intervals (95%) based on 5,000 resamples. Results of this mediation analysis are presented in detail in Table 5.

**Table 5:** Indirect effects of ATA in the association between PCICT classes and multidimensional health outcomes.

Category	Total effect	Direct effect	Indirect effect	Boot SE	LLCI	ULCI
PCICT–ATA–SRH						
All-domains beneficiaries	0.281^***^	0.246^***^	0.034^***^	0.006	0.022	0.047
Foundational-domains beneficiaries	0.173^***^	0.152^***^	0.021^***^	0.006	0.010	0.033
Extended-domains frustrated	0.017	0.004	0.013^*^	0.002	0.004	0.024
Compound experience	0.020	0.031	−0.011^*^	0.006	−0.022	−0.000
PCICT–ATA–DS						
All-domains beneficiaries	−1.295^***^	−1.185^***^	−0.110^***^	0.022	−0.154	−0.066
Foundational-domains beneficiaries	−0.661^***^	−0.592^***^	−0.069^***^	0.019	−0.107	−0.030
Extended-domains frustrated	0.824^***^	0.867^***^	−0.043^*^	0.018	−0.079	−0.006
Compound experience	−0.032	−0.069	0.037^*^	0.018	0.001	0.072
PCICT–ATA–LS						
All-domains beneficiaries	0.290^***^	0.270^***^	0.020^***^	0.005	0.011	0.029
Foundational-domains beneficiaries	0.132^***^	0.120^***^	0.012^**^	0.004	0.005	0.020
Extended-domains frustrated	0.121^***^	0.113^***^	0.008^*^	0.003	0.001	0.015
Compound experience	0.085^***^	0.092^***^	−0.007^*^	0.003	−0.013	−0.000
PCICT–ATA–SA						
All-domains beneficiaries	2.584^***^	2.354^***^	0.230^***^	0.040	0.150	0.310
Foundational-domains beneficiaries	0.731^***^	0.586^***^	0.145^***^	0.039	0.069	0.220
Extended-domains frustrated	0.814^***^	0.724^***^	0.090^*^	0.038	0.016	0.164
Compound experience	0.943^***^	1.020^***^	−0.077^*^	0.037	−0.150	−0.003

Reference group, Unaffected; Boot, Bootstrap; PCICT, Perceived convenience of information and communication technology, ATA, Attitudes toward aging, SRH, Self-rated health, DS, Depressive symptoms, LS, Life satisfaction, SA, Social adaptation; Boot SE, bootstrap standard error; LLCI, lower limit of the 95% confidence interval; ULCI, upper limit of the 95% confidence interval. *p < 0.05, **p < 0.01, ***p < 0.001.

Significant indirect associations through ATA were observed for most PCICT classes and health outcomes. For the “All-domains Beneficiaries” and “Foundational-domains Beneficiaries” groups, significant indirect effects were found across all health outcomes, consistently pointing to better health. The pattern differed for the “Extended-domains Frustrated” group. For depressive symptoms, ATA partly offset the positive association between this group and depressive symptoms. Nevertheless, the “Compound Experience” class showed an opposite pattern of indirect effects through ATA. For both life satisfaction and social adaptation, significant negative indirect associations through ATA were observed. This suggests that the group’s mixed digital experiences were associated with less positive ATA, which was, in turn, associated with poorer self-rated health, more severe depressive symptoms, lower life satisfaction, and poorer social adaptation. This pattern differed from the beneficial indirect effects observed in the other classes.

### 4.7 Sensitivity analysis

We conducted two sensitivity analyses to examine the robustness of our findings. First, we repeated all models using stabilized inverse probability-of-inclusion weights to assess whether complete-case selection affected the results. The differences between included and excluded participants were generally small (Supplementary Table S2), and weighting did not materially change the five-class solution or the main findings (Supplementary Figure S1, Supplementary Tables S3–S6). Second, we examined the role of internet use frequency. Internet use frequency was closely associated with PCICT class membership but did not correspond one-to-one with the latent classes (Supplementary Table S7). After additionally adjusting for internet use frequency, the overall associations between PCICT classes and health outcomes and the indirect associations through ATA remained consistent with the main analyses (Supplementary Tables S8, S9). These results validated the robustness of our conclusions to some extent.

## 5 Discussion

Based on the latest nationally representative dataset and using the person-centered LCA method, this study aimed to identify different classes of PCICT among Chinese older adults. Our analysis successfully identified five distinct classes of PCICT, highlighting that the ICT experiences of older adults are heterogeneous. This study expanded on previous work by identifying potential factors related to these classes and examining how these classes were differentially associated with multidimensional health outcomes. In addition, this study found that ATA partly accounted for the associations between PCICT classes and health outcomes.

### 5.1 PCICT classes

We identified five PCICT classes: “All-domains Beneficiaries,” “Foundational-domains Beneficiaries,” “Extended-domains Frustrated,” “Compound Experience,” and “Unaffected.” Our results extend beyond previous studies that focus on the first and second digital divides by empirically exploring the third digital divide (the benefit gap) from a perceived convenience perspective (4). By identifying these five PCICT classes, our findings underscore the significant diversity in how older adults perceive the convenience of ICT. Although previous studies have acknowledged the benefits of internet technology (3, 72, 94), no research so far has reported the overall classes that the current study identified. The “All-domains Beneficiaries” class was characterized by perceived convenience across all eight life domains. In contrast, the “Unaffected” class was characterized predominantly by “No impact” or “Uncertain” responses across these domains, suggesting that participants in this class generally did not perceive a clear impact of ICT on their daily lives. However, given the high proportion of non-users in this class, this response pattern may also partly reflect limited exposure to ICT. Between these two classes were three more nuanced classes. Participants in the “Foundational-domains Beneficiaries” class mainly perceived convenience in basic areas of daily life, such as social interaction and shopping. In extended areas, including health services and learning, they were more likely to report “No impact.” This pattern may be related to differences in how familiar older adults are with ICT applications, as some may be more familiar with its use for social communication, information access, and entertainment than for extended applications such as health services and learning (27, 95). The “Extended-domains Frustrated” class reported convenience in foundational domains but inconvenience in extended domains. Previous studies suggest that complex menus, poorly designed interfaces, misinformation, and information overload may hinder older adults’ use of digital services and contribute to frustration (96, 97). The “Compound Experience” class was the largest group. Participants in this group showed no dominant response pattern across eight life domains.

### 5.2 Factors associated with PCICT class

Based on the findings from the PCICT classes, this study further investigated the associations between PCICT classes and individual, family, and community factors. At the individual level, age, education level, marital status, and annual income were significantly related to PCICT classes. For example, individuals aged 60–69, with a spouse, with higher education, and with higher income were more likely to belong to the “All-domains Beneficiaries” class, which is consistent with previous research (4, 34). This further underscores the association between socioeconomic resources and digital inequality (98, 99).

Older adults with chronic conditions were less likely to belong to the “All-domains Beneficiaries” and “Compound Experience” classes, but more likely to belong to the “Foundational-domains Beneficiaries” class. One possible explanation is that chronic conditions may limit physical mobility and increase older adults’ reliance on ICT for essential daily tasks. This may enable them to benefit more from ICT use (84, 100), while health-related limitations may also make more complex digital applications difficult to use.

The associations between family factors and older adults’ PCICT class were particularly complex. Family co-residence was related to PCICT class membership in different ways. Older adults living with family were more likely to belong to the “Foundational-domains Beneficiaries” and “Extended-domains Frustrated” classes, but not to the “All-domains Beneficiaries” class. This may suggest that although family support is crucial for initial access and basic use (34), it may not be sufficient for the “All-domains Beneficiaries” class. One possible interpretation is that the available support may be sufficient for simple tasks but insufficient for more complex ones, and greater reliance on family assistance may coexist with frustration in extended domains. A related finding was that having more children was associated with a lower likelihood of belonging to the “All-domains Beneficiaries” class. Taken together, these findings highlight the important difference between the quantity and quality of family support. Living with family or having more children may give older adults greater access to help with digital tasks. Previous studies describe this as “proxy internet use,” in which family members use or navigate digital services on behalf of older adults, often because doing so is quicker or more convenient (101, 102). According to Self-Determination Theory (103), this substitutive support may undermine older adults’ fundamental psychological needs for autonomy and competence. This may help explain the observed associations of co-residence and number of children with different PCICT classes.

Place of residence was also related to PCICT class membership. Urban older adults were more likely than their rural counterparts to belong to the “All-domains Beneficiaries” and “Foundational-domains Beneficiaries” classes. Previous studies have also found that older adults in urban areas benefit more from superior infrastructure and a well-developed ecosystem of internet-enabled services (104–106). Bronfenbrenner’s Ecological Systems Theory offers a compelling framework for understanding this disparity. The theory posits that individual outcomes are shaped by external environmental systems (107). In this context, the urban environment functions as a highly supportive exosystem, where superior infrastructure and a rich ecosystem of services create favorable conditions for older adults to obtain benefits from ICT use. Conversely, the lack of a supportive exosystem in many rural areas may constrain the benefits available from digital technology and be associated with inequalities in outcomes.

In addition, our findings highlight significant regional disparities in the distribution of latent classes. Compared to older adults living in the western region, those in the central and eastern regions were more likely to fall into the “Foundational-domains Beneficiaries” class, but less likely to be classified into the “All-domains Beneficiaries,” “Extended-domains Frustrated,” or “Compound Experience” classes. This pattern may indicate that, in the central and eastern regions, which are relatively more developed economically and socially, residents tend to enjoy widespread benefits in these core life areas. However, they still face challenges in achieving comprehensive improvements in higher-level “extended domains,” such as health services and learning and training. In contrast, the findings indicate that the western region may display a more polarized pattern. While some groups manage to achieve benefits across all domains, others are more susceptible to experiencing extended-domain frustrations or compound disadvantages. These results may reflect uneven regional development in China. The eastern and central regions generally have a stronger economic foundation and more advanced digital infrastructure. These conditions may support convenient online shopping, entertainment, and access to information and may be associated with fewer difficulties in foundational life domains. Older adults in these regions may still find it difficult to benefit from ICT in extended domains, partly because high-quality healthcare, financial, and other services are not equally available or easy to access. In western China, policy support and the concentration of certain resources may create digital opportunities for some groups. Others, however, may continue to face limited access to services and persistent digital disadvantages.

### 5.3 The relationship between PCICT classes and health outcomes

This study provides evidence that PCICT classes were differentially associated with health outcomes. Specifically, both “All-domains Beneficiaries” and “Foundational-domains Beneficiaries” classes were associated with more favorable outcomes across all health indicators, when compared to the “Unaffected” class. Previous studies have also linked ICT-related benefits with physical, mental, and social health through social connections, access to health information, entertainment resources, and greater convenience in daily life (3, 8, 43, 108). However, reverse directionality is also plausible. For instance, older adults with better functional ability, vision, and learning capacity perceived greater benefits from digital health and social services (109), suggesting that better health and functioning may enable older adults to engage with ICT more effectively and perceive greater convenience and benefits from its use.

We also found that the “Extended-domains Frustrated” class was linked to more severe depressive symptoms. This association has received limited attention in previous studies. This result suggests that although the “Extended-domains Frustrated” class reported higher life satisfaction and better social adaptation, it also showed more severe depressive symptoms than the “Unaffected” class. This finding expands the current literature, which highlights the potential risks linked to ICT. According to the Stress Process Model (45, 110), when older adults try to use advanced digital services (such as online health services or online learning) but find that they are poorly designed or beyond their skill level, the gap between expectations and reality may be perceived as a chronic stressor. Within the Stress Process Model, repeated failure or inconvenience may be associated with lower self-efficacy, negative emotions, and more severe depressive symptoms (111), although the temporal ordering of these factors cannot be determined from the present data. Conversely, previous evidence suggests a reciprocal relationship between depressive symptoms and internet engagement, indicating that older adults with more severe depressive symptoms may be less inclined to engage with ICT (112), and may be more likely to perceive its use as inconvenient or frustrating.

Additionally, psychological research suggests that technology comes with a psychological cost to the user; frustrating experiences during ICT use have a negative impact on a user’s psychological well-being (113). Therefore, individuals in the “Extended-domains Frustrated” class may experience more negative feelings, such as depressive symptoms, when they encounter frustrating ICT experiences. Compared with the “Unaffected” class, the “Compound Experience” class showed higher life satisfaction and better social adaptation, but no differences in physical health or depressive symptoms. Thus, the mixed experiences of this group were not consistently related to all dimensions of health. Their ICT use may have provided some benefits, but also confused them, with the positive and negative effects balancing each other, which may help explain the insignificant differences in physical and mental health indicators.

### 5.4 The mediating role of ATA

The mediation results showed that ATA partly accounted for the observed associations between PCICT classes and health outcomes. This suggests that the third-level digital divide may be related to health not only through differences in the perceived benefits of ICT, but also through older adults’ ATA. In line with Stereotype Embodiment Theory (56), our findings suggest that digital experiences may be related to older adults’ ATA. Older adults in the “All-domains Beneficiaries” and “Foundational-domains Beneficiaries” classes may gain a sense of autonomy, competence, and social inclusion (67, 68). This process may be associated with more positive ATA, which may, in turn, be associated with better health outcomes. For the “Extended-domains Frustrated” class, ATA may partially account for the association between a frustrating digital experience and depressive symptoms, which is consistent with the Stress Process Model (45, 110). Within the framework of the Stress Process Model, ATA may function as a crucial psychological resource that helps explain the association between chronic strain (frustrating digital experiences) and depressive symptoms. For the “Compound Experience” class, mixed digital experiences were associated with more negative ATA, which was in turn associated with lower life satisfaction and poorer social adaptation. For this group, compound digital experience may highlight their limitations more than their capabilities, and may be associated with a greater sense of being left behind and stronger perceptions of social marginalization (48).

### 5.5 Strengths and limitations

This study has several notable strengths. First, it is among the first studies to use a person-centered LCA to examine differences in older adults’ PCICT. The analysis identified five distinct classes, showing that older adults vary considerably in how they perceive the convenience of ICT across different areas of daily life. Second, unlike previous studies that mainly focus on the “first digital divide” and “second digital divide,” this study directly addresses the “third digital divide” by exploring the benefit gap, making a significant contribution to the digital divide literature. Third, this study utilized a large-scale, nationally representative dataset, which enhances the generalizability of our findings. Lastly, a key contribution of this study is its comprehensive, multi-stage analytical approach. We first moved beyond simple usage metrics to identify these distinct classes of PCICT using LCA. We then investigated the characteristics associated with these classes. This study also examined the potential mediating role of ATA in the associations between PCICT classes and health outcomes. The results showed that ATA partly accounted for these associations.

This study also has several limitations. First, this study relies on self-reported data, which may introduce social desirability bias. Future studies could combine self-reports with objective measures or reports from family members. Second, the cross-sectional data do not allow us to determine the temporal or causal direction of the associations. Although PCICT was related to health, reverse relationships are also possible. Older adults in better physical or mental health may be more likely to perceive greater convenience from ICT. Similarly, the analytical placement of ADL limitations and chronic conditions as factors associated with class membership and self-rated health as a health outcome reflects their different analytical roles in the present study rather than an established temporal ordering. Self-rated health may also be associated with PCICT class membership, while digital experiences may in turn be related to functional status or chronic disease management. Accordingly, the association between PCICT and health outcomes and the observed indirect associations through ATA should not be interpreted as evidence of causal relationships or mechanisms. Longitudinal studies are needed to establish the temporal ordering among PCICT, ATA, and health outcomes and to clarify how these factors may be related to one another over time. Third, PCICT was measured using the eight items included in the CLASS 2023 questionnaire. Although these items covered both foundational and extended life domains, they may not capture all domains in which older adults perceive convenience, inconvenience, or other impacts of ICT. Therefore, the identified latent class structure should be interpreted in light of the content coverage of the questionnaire. Future studies could examine additional ICT-related areas of daily life and improve the measurement of PCICT. The five-class solution should also be tested in other samples. Fourth, although this study assessed health across physical, mental, and social dimensions based on the WHO framework, these measures do not capture all aspects of health in later life. Other dimensions, such as cognitive health, were beyond the scope of the present study and should be considered in future research. In addition, some participants were excluded because of missing data. Although the weighted analysis produced similar results, selection bias due to unmeasured differences between included and excluded participants cannot be ruled out. Future studies could use data with fewer missing values and compare different approaches to missing data, such as multiple imputation and full-information maximum likelihood.

## 6 Conclusion

Utilizing LCA, this study identified five classes of PCICT among Chinese older adults. The “Compound Experience” class accounted for the largest share of the sample, whereas the “Extended-domains Frustrated” class was the least common. Multinomial logistic regression showed that older adults’ individual characteristics, family characteristics, and community factors were related to PCICT classes. Additionally, the study found that both the “All-domains Beneficiaries” and “Foundational-domains Beneficiaries” classes were associated with more favorable health outcomes across all indicators. Older adults in the “Extended-domains Frustrated” class reported more depressive symptoms than those in the “Unaffected” class. ATA partly accounted for the associations between PCICT classes and health outcomes, although the direction of the indirect effects differed across classes. Our findings may help inform more tailored digital support for older adults.

## Data Availability

The datasets presented in this article are not readily available because the authors have no rights to share this data. This study uses open data. Researchers can apply for and download the data from the website (https://cssd.ruc.edu.cn/home). Requests to access the datasets should be directed to https://cssd.ruc.edu.cn/home.
