# Supplementary material for: The relationships between perceived ICT convenience patterns, attitudes toward aging, and multidimensional health outcomes among older adults
Source: Front Public Health. 2026 Sep 9;14:1955930. doi: 10.3389/fpubh.2026.1955930 (PMC13598962; doi:10.3389/fpubh.2026.1955930)
Supplement: Supplementary file 1 [file Supplementary_file_1.docx]

**Supplementary Material**

**Table S1.** Descriptive statistics of demographic distribution characteristics across the five classes.

|  | All-domains Beneficiaries | Foundational-domains Beneficiaries | Extended-domains Frustrated | Compound  Experience | Unaffected | χ^2^ -Test/ Anova |
| --- | --- | --- | --- | --- | --- | --- |
|  | 2,156 (21.73%) | 1,887 (19.02%) | 1,266 (12.76%) | 3,146 (31.71%) | 1,465 (14.77%) |  |
| Age |  |  |  |  |  |  |
| 60-69 years old | 1,145 (53%) | 949 (50%) | 594 (47%) | 1,010 (32%) | 329 (22%) | <0.001 |
| 70-79 years old | 841 (39%) | 815 (43%) | 553 (44%) | 1,835 (58%) | 792 (54%) |  |
| 80 years old and above | 170 (8%) | 123 (7%) | 119 (9%) | 301 (10%) | 344 (23%) |  |
| Gender |  |  |  |  |  |  |
| Female | 1,000 (46%) | 876 (46%) | 593 (47%) | 1,575 (50%) | 754 (51%) | 0.002 |
| Male | 1,156 (54%) | 1,011 (54%) | 673 (53%) | 1,571 (50%) | 711 (49%) |  |
| Education level |  |  |  |  |  |  |
| Illiterate | 232 (11%) | 216 (11%) | 187 (15%) | 605 (19%) | 469 (32%) | <0.001 |
| Primary education or below | 688 (32%) | 744 (39%) | 545 (43%) | 1,359 (43%) | 709 (48%) |  |
| Junior high school or above | 1,236 (57%) | 927 (49%) | 534 (42%) | 1,182 (38%) | 287 (20%) |  |
| Marital status |  |  |  |  |  |  |
| Other | 243 (11%) | 207 (11%) | 181 (14%) | 684 (22%) | 403 (28%) | <0.001 |
| Have a spouse | 1,913 (89%) | 1,680 (89%) | 1,085 (86%) | 2,462 (78%) | 1,062 (72%) |  |
| Chronic status |  |  |  |  |  |  |
| No | 632 (29%) | 262 (14%) | 233 (18%) | 741 (24%) | 204 (14%) | <0.001 |
| Yes | 1,524 (71%) | 1,625 (86%) | 1,033 (82%) | 2,405 (76%) | 1,261 (86%) |  |
| Annual income (Ln) | 9.76 (1.06) | 9.82 (1.04) | 9.58 (1.13) | 9.42 (1.06) | 8.87 (1.18) | <0.001 |
| Activities of daily living | 6.08 (0.59) | 6.14 (0.67) | 6.08 (0.43) | 6.18 (0.96) | 6.21 (1.20) | 0.209 |
| Number of children | 1.73 (1.04) | 1.93 (1.04) | 2.08 (1.07) | 2.25 (1.16) | 2.44 (1.26) | <0.001 |
| Living arrangement |  |  |  |  |  |  |
| Live with family | 2,040 (95%) | 1,772 (94%) | 1,162 (92%) | 2,810 (89%) | 1,297 (89%) | <0.001 |
| Live alone | 116 (5%) | 115 (6%) | 104 (8%) | 336 (11%) | 168 (11%) |  |
| Residence |  |  |  |  |  |  |
| Rural | 827 (38%) | 813 (43%) | 674 (53%) | 1,894 (60%) | 975 (67%) | <0.001 |
| Urban | 1,329 (62%) | 1,074 (57%) | 592 (47%) | 1,252 (40%) | 490 (33%) |  |
| Region |  |  |  |  |  |  |
| Eastern areas | 452 (21%) | 652 (35%) | 439 (35%) | 1,249 (40%) | 582 (40%) | <0.001 |
| Central areas | 1,150 (53%) | 950 (50%) | 479 (38%) | 955 (30%) | 424 (29%) |  |
| Western areas | 554 (26%) | 285 (15%) | 348 (27%) | 942 (30%) | 459 (31%) |  |

**Sensitivity analysis 1**

To assess the potential risk of selection bias arising from the exclusion of participants with missing data, we conducted a series of sensitivity analyses. First, we compared the observed characteristics of the 9,920 participants included in the analytical sample with those of 1,692 excluded participants who had sufficient observed information on the covariates used in the comparison. The remaining 36 excluded participants could not be included in this comparison because of incomplete covariate information. Pearson’s chi-square tests were used for categorical variables, Kruskal-Wallis tests were used for continuous variables (Kruskal & Wallis, 1952), and standardized mean differences (SMDs) were calculated to evaluate the magnitude of between-group differences. An absolute SMD below 0.10 was considered indicative of a small difference (Austin, 2009).

Second, among the 11,612 participants with complete information on all covariates used in the inclusion model (9,920 included participants and 1,692 excluded participants), a logistic regression model was used to estimate each participant’s probability of inclusion in the complete-case sample based on age, gender, education level, marital status, chronic condition, annual income, activities of daily living, number of children, living arrangement, residence, and region. Stabilized inverse probability-of-inclusion weights were calculated as the marginal probability of inclusion divided by the predicted probability of inclusion.

Finally, the five-class LCA, multinomial logistic regression, health-outcome regression models, and KHB mediation models were re-estimated among the 9,920 participants in the analytical sample using the stabilized weights. Robust standard errors were used in the weighted regression and mediation analyses, and the indirect effects in the weighted mediation models were evaluated using 95% Wald confidence intervals.

Table S2 compares the observed characteristics of participants included in the analytical sample with those excluded because of missing variables. Most standardized mean differences were below 0.10, indicating generally small differences between the two groups. Modest differences were observed for annual income and region, with region showing the largest imbalance (SMD = 0.169) (Table S2). Overall, the observed differences were generally limited, although the potential influence of complete-case selection could not be entirely ruled out. Therefore, inverse probability-of-inclusion weighting was further applied to assess whether complete-case selection materially affected the main findings.

Table S2. Comparison of characteristics between included participants and excluded participants with sufficient covariate information.

| Variables | Category (Range) | Included Sample (N=9920) | Excluded Sample (N=1692) | SMD | *P* |
| --- | --- | --- | --- | --- | --- |
| Age | 60-69 years old | 4,027 (40.59%) | 675 (39.89%) | 0.069 | 0.017 |
|  | 70-79 years old | 4,836 (48.75%) | 871 (51.48%) |  |  |
|  | 80 years old and above | 1,057 (10.66%) | 146 (8.63%) |  |  |
| Gender | Female | 4,798 (48.37%) | 794 (46.93%) | 0.029 | 0.273 |
|  | Male | 5,122 (51.63%) | 898 (53.07%) |  |  |
| Education level | Illiterate | 1,709 (17.23%) | 296 (17.49%) | 0.076 | 0.011 |
|  | Primary education or below | 4,045 (40.78%) | 627 (37.06%) |  |  |
|  | Junior high school or above | 4,166 (42.00%) | 769 (45.45%) |  |  |
| Marital status | Have a spouse | 8,202 (82.68%) | 1,450 (85.70%) | 0.083 | 0.002 |
|  | Others | 1,718 (17.32%) | 242 (14.30%) |  |  |
| Chronic condition | No | 2,072 (20.89%) | 400 (23.64%) | 0.066 | 0.011 |
|  | Yes | 7,848 (79.11%) | 1,292 (76.36%) |  |  |
| Annual income (Ln) | 4.61~12.89 | 9.51 (1.13) | 9.62 (1.08) | 0.105 | <0.001 |
| Activities of daily living | 6~18 | 6.14 (0.83) | 6.19 (0.99) | 0.050 | 0.520 |
| Number of children | 0~9 | 2.08 (1.14) | 2.05 (1.25) | 0.024 | 0.114 |
| Living arrangement | Live with family | 9,081 (91.54%) | 1,591 (94.03%) | 0.096 | <0.001 |
|  | Live alone | 839 (8.46%) | 101 (5.97%) |  |  |
| Residence | Rural | 5,183 (52.25%) | 930 (54.96%) | 0.054 | 0.039 |
|  | Urban | 4,737 (47.75%) | 762 (45.04%) |  |  |
| Region | Central areas | 3,374 (34.01%) | 669 (39.54%) | 0.169 | <0.001 |
|  | Eastern areas | 3,958 (39.90%) | 701 (41.43%) |  |  |
|  | Western areas | 2,588 (26.09%) | 322 (19.03%) |  |  |

*Note*. Values are presented as n (%) for categorical variables and mean (SD) for continuous variables. SMD = standardized mean difference. For categorical variables with more than two categories, the maximum absolute SMD across categories is reported. P values were obtained using Pearson’s chi-square tests for categorical variables and Kruskal–Wallis tests for continuous variables. Of the 1,728 participants excluded from the primary analysis, 1,692 had sufficient information on all covariates included in this comparison; the remaining 36 were not included because of incomplete covariate information.

The inverse probability-weighted LCA reproduced the same five conditional item-response probability patterns identified in the primary analysis (Figure S1). The distribution of participants’ most likely class membership was also highly similar between the two analyses, with absolute differences of less than 0.20 percentage points across all five classes (Table S3).

Figure S1. Conditional item-response probabilities for the five PCICT classes in the inverse probability-weighted analysis

Table S3. Comparison of estimated class prevalences between the primary and inverse probability-weighted analyses.

| PCICT classes | Primary analysis (%) | IPW analysis (%) | Difference |
| --- | --- | --- | --- |
| All-domains Beneficiaries | 2,156 (21.73) | 2,174 (21.92) | +0.19 |
| Foundational-domains Beneficiaries | 1,887 (19.02) | 1,868 (18.83) | -0.19 |
| Extended-domains Frustrated | 1,266 (12.76) | 1,258 (12.68) | -0.08 |
| Compound Experience | 3,146 (31.71) | 3,140 (31.65) | -0.06 |
| Unaffected | 1,465 (14.77) | 1,480 (14.92) | +0.15 |

*Note.* Values are presented as n (%), based on participants’ most likely latent class membership. Class labels and ordering in the inverse probability-weighted analysis were aligned with those used in the primary analysis. Differences were calculated as the percentage in the IPW analysis minus that in the primary analysis and are expressed in percentage points. IPW = inverse probability weighting.

The inverse probability-weighted multinomial logistic regression (Table S4) produced patterns that were highly consistent with those observed in the primary analysis. Although the statistical significance of a few associated factors showed minor fluctuations, the directions and overall significance patterns of the associations remained largely unchanged, suggesting that the findings were not materially altered by adjustment for observed differences associated with complete-case selection.

Table S4. Factors associated with PCICT class membership in the inverse probability-weighted analysis.

| VARIABLES | All-domains Beneficiaries | | | Foundational-domains Beneficiaries | | Extended-domains Frustrated | | Compound Experience |
| --- | --- | --- | --- | --- | --- | --- | --- | --- |
| ***Individual-level factors*** |  | | |  | |  | |  |
| Age (Ref. 60-69 years old) | |  |  | |  | |  | |
| 70-79 years old | 0.472^***^ | | | 0.463^***^ | | 0.488^***^ | | 0.912 |
|  | (0.400 - 0.558) | | | (0.391 - 0.549) | | (0.407 - 0.586) | | (0.779 - 1.068) |
| 80 years old and above | 0.382^***^ | | | 0.242^***^ | | 0.330^***^ | | 0.376^***^ |
|  | (0.291 - 0.502) | | | (0.182 - 0.320) | | (0.246 - 0.442) | | (0.296 - 0.477) |
| Gender (Ref. Female) | 1.019 | | | 1.049 | | 1.006 | | 0.886 |
| Male | (0.880 - 1.180) | | | (0.905 - 1.216) | | (0.858 - 1.181) | | (0.777 - 1.010) |
| Education level (Ref. Illiterate) | |  |  | |  | |  | |
| Primary education or below | 1.556^***^ | | | 1.723^***^ | | 1.646^***^ | | 1.367^***^ |
|  | (1.268 - 1.910) | | | (1.401 - 2.119) | | (1.330 - 2.037) | | (1.165 - 1.603) |
| Junior high school or above | 3.520^***^ | | | 2.861^***^ | | 2.714^***^ | | 2.668^***^ |
|  | (2.774 - 4.467) | | | (2.236 - 3.661) | | (2.105 - 3.501) | | (2.163 - 3.290) |
| Marital status (Ref. Other) | 1.458^**^ | | | 1.820^***^ | | 1.539^**^ | | 0.992 |
| Have a spouse | (1.134 - 1.873) | | | (1.410 - 2.347) | | (1.184 - 2.000) | | (0.817 - 1.204) |
| Chronic condition (Ref. No) | 0.594^***^ | | | 1.443^***^ | | 0.953 | | 0.586^***^ |
| Yes | (0.494 - 0.715) | | | (1.169 - 1.781) | | (0.768 - 1.182) | | (0.492 - 0.697) |
| Annual income (Ln) | 1.654^***^ | | | 1.772^***^ | | 1.580^***^ | | 1.482^***^ |
|  | (1.544 - 1.772) | | | (1.653 - 1.900) | | (1.472 - 1.696) | | (1.397 - 1.573) |
| Activities of daily living | 0.987 | | | 1.066 | | 0.915 | | 1.067^*^ |
|  | (0.889 - 1.095) | | | (0.983 - 1.156) | | (0.822 - 1.019) | | (1.000 - 1.139) |
| ***Family-level factors*** |  | | |  | |  | |  |
| Number of children | 0.861^***^ | | | 1.003 | | 1.021 | | 1.053 |
|  | (0.800 - 0.926) | | | (0.932 - 1.080) | | (0.947 - 1.100) | | (0.991 - 1.119) |
| Living arrangement (Ref. Live alone) | 1.117 | | | 1.589^**^ | | 1.482^*^ | | 1.278 |
| Live with family | (0.798 - 1.565) | | | (1.138 - 2.219) | | (1.056 - 2.081) | | (0.992 - 1.646) |
| ***Community-level factors*** |  | | |  | |  | |  |
| Residence (Ref. Rural) | 1.516^***^ | | | 1.347^***^ | | 1.098 | | 0.886 |
| Urban | (1.285 - 1.788) | | | (1.136 - 1.598) | | (0.921 - 1.308) | | (0.760 - 1.033) |
| Region (Ref. Western areas) |  | | |  | |  | |  |
| Central areas | 0.468^***^ | | | 1.341^**^ | | 0.800^*^ | | 0.838^*^ |
|  | (0.387 - 0.566) | | | (1.100 - 1.636) | | (0.655 - 0.976) | | (0.717 - 0.980) |
| Eastern areas | 0.783^*^ | | | 1.483^***^ | | 0.763^*^ | | 0.597^***^ |
|  | (0.641 - 0.957) | | | (1.193 - 1.843) | | (0.616 - 0.945) | | (0.495 - 0.720) |
| Constant | 0.002^***^ | | | 0.000^***^ | | 0.007^***^ | | 0.120^***^ |
|  | (0.001 - 0.006) | | | (0.000 - 0.001) | | (0.002 - 0.020) | | (0.053 - 0.270) |
| Observations | 9,920 | | | 9,920 | | 9,920 | | 9,920 |
| Pseudo R^2^ | 0.090 | | | 0.090 | | 0.090 | | 0.090 |

*Note.* The “Unaffected” class was used as the reference outcome. The model was estimated using stabilized inverse probability-of-inclusion weights and adjusted for the same covariates as the primary analysis. The estimates reported in Table S4 are odds ratios. Ref. = reference category; values in parentheses are 95% confidence intervals. ^*^*p* < 0.05, ^**^*p* < 0.01, ^***^*p* < 0.001.

The inverse probability-weighted estimates (Table S5) were highly consistent with those obtained in the primary analysis. The directions and statistical significance of all associations remained unchanged, and the minor changes in coefficient magnitude did not alter the interpretation of the findings.

Table S5. Associations between PCICT classes and health outcomes in the inverse probability-weighted analysis

| VARIABLES | SRH | DS | LS | SA |
| --- | --- | --- | --- | --- |
| PCICT (Ref. Unaffected) |  |  |  |  |
| All-domains Beneficiaries | 0.246^***^ | -1.141^***^ | 0.268^***^ | 2.341^***^ |
|  | (0.024) | (0.107) | (0.024) | (0.133) |
| Foundational-domains Beneficiaries | 0.155^***^ | -0.572^***^ | 0.117^***^ | 0.582^***^ |
|  | (0.024) | (0.113) | (0.025) | (0.130) |
| Extended-domains Frustrated | -0.002 | 0.910^***^ | 0.110^***^ | 0.684^***^ |
|  | (0.028) | (0.122) | (0.026) | (0.132) |
| Compound Experience | 0.032 | -0.053 | 0.090^***^ | 1.001^***^ |
|  | (0.023) | (0.098) | (0.024) | (0.124) |
| Control variables | YES | YES | YES | YES |
| Constant | 3.985^***^ | 14.818^***^ | 3.603^***^ | 20.971^***^ |
|  | (0.118) | (0.449) | (0.109) | (0.586) |
| Observations | 9,920 | 9,920 | 9,920 | 9,920 |
| R^2^ | 0.182 | 0.184 | 0.095 | 0.124 |
| F | 112.8 | 121.6 | 63.50 | 68.40 |

*Note.* Standard errors in parentheses, ^***^ *p*<0.001, ^**^ *p*<0.01, ^*^ *p*<0.05, SRH = Self-rated health, DS = Depressive symptoms, LS = Life satisfaction, SA = Social adaptation.

The inverse probability-weighted mediation analysis (Table S6) also reproduced the indirect-effect patterns observed in the primary analysis. All indirect effects retained the same directions and statistical significance, with only minimal changes in magnitude, indicating that the mediating role of ATA remained robust after weighting.

Table S6. Mediation effects of ATA in the inverse probability-weighted analysis.

| Category | Total  effect | Direct  effect | Indirect  effect | Robust SE | LLCI | ULCI |
| --- | --- | --- | --- | --- | --- | --- |
| **PCICT - ATA- SRH** |  |  |  |  |  |  |
| All-domains Beneficiaries | 0.280^***^ | 0.246^***^ | 0.034^***^ | 0.006 | 0.022 | 0.047 |
| Foundational-domains Beneficiaries | 0.177^***^ | 0.155^***^ | 0.021^***^ | 0.006 | 0.010 | 0.032 |
| Extended-domains Frustrated | 0.011 | -0.002 | 0.013^*^ | 0.005 | 0.003 | 0.024 |
| Compound Experience | 0.020 | 0.032 | -0.012^*^ | 0.005 | -0.022 | -0.001 |
| **PCICT - ATA- DS** |  |  |  |  |  |  |
| All-domains Beneficiaries | -1.251^***^ | -1.141^***^ | -0.109^***^ | 0.022 | -0.153 | -0.066 |
| Foundational-domains Beneficiaries | -0.639^***^ | -0.572^***^ | -0.067^***^ | 0.019 | -0.104 | -0.030 |
| Extended-domains Frustrated | 0.868^***^ | 0.910^***^ | -0.042^*^ | 0.018 | -0.077 | -0.008 |
| Compound Experience | -0.016 | -0.053 | 0.037^*^ | 0.017 | 0.003 | 0.071 |
| **PCICT - ATA- LS** |  |  |  |  |  |  |
| All-domains Beneficiaries | 0.289^***^ | 0.268^***^ | 0.020^***^ | 0.005 | 0.011 | 0.029 |
| Foundational-domains Beneficiaries | 0.129^***^ | 0.117^***^ | 0.012^**^ | 0.004 | 0.005 | 0.020 |
| Extended-domains Frustrated | 0.117^***^ | 0.110^***^ | 0.008^*^ | 0.003 | 0.001 | 0.014 |
| Compound Experience | 0.083^**^ | 0.090^***^ | -0.007^*^ | 0.003 | -0.013 | -0.000 |
| **PCICT - ATA- SA** |  |  |  |  |  |  |
| All-domains Beneficiaries | 2.576^***^ | 2.341^***^ | 0.234^***^ | 0.041 | 0.154 | 0.314 |
| Foundational-domains Beneficiaries | 0.726^***^ | 0.582^***^ | 0.144^***^ | 0.038 | 0.070 | 0.217 |
| Extended-domains Frustrated | 0.775^***^ | 0.684^***^ | 0.090^*^ | 0.036 | 0.019 | 0.161 |
| Compound Experience | 0.922^***^ | 1.001^***^ | -0.079^*^ | 0.036 | -0.150 | -0.009 |

*Note.* Reference group= Unaffected; Estimates were obtained from inverse probability-weighted KHB models with robust standard errors; PCICT = Perceived convenience of information and communication technology, ATA = Attitudes toward aging, SRH = Self-rated health, DS = Depressive symptoms, LS = Life satisfaction, SA = Social adaptation; ^*^*p* < 0.05, ^**^*p* < 0.01, ^***^*p* < 0.001. Robust SE = robust standard error; LLCI = lower limit of the 95% confidence interval; ULCI = upper limit of the 95% confidence interval.

**Sensitivity analysis 2**

We first examined the distribution of internet use frequency across PCICT classes. In the original measure, internet use frequency was classified into five categories: no internet use (47.31%), yearly use (0.51%), monthly use (1.27%), weekly use (8.76%), and daily use (42.15%). Because the yearly and monthly categories contained relatively few participants, we combined the three non-daily use categories (yearly, monthly, and weekly use) to improve the stability and interpretability of the analysis. Internet use frequency was therefore recoded into three categories: non-user (47.31%), non-daily user (10.54%), and daily user (42.15%). The same three-category measure was used to compare internet use frequency across PCICT classes and in the subsequent analyses.

The distribution of internet use frequency across PCICT classes is presented in Table S7. Internet use frequency was significantly associated with PCICT class membership (χ²(8) = 3,587.41, *p* < 0.001; Cramér’s V = 0.425). Daily internet use was most prevalent among the All-domains Beneficiaries (78.62%), whereas the Unaffected class consisted predominantly of non-users (89.35%). However, internet use frequency did not correspond one-to-one with PCICT class membership. The Unaffected class also included non-daily users (3.89%) and daily users (6.76%), while the All-domains Beneficiaries included non-users (15.72%) and non-daily users (5.66%). Variation in internet use frequency was also observed within the other PCICT classes. These results indicate that internet use frequency is closely associated with PCICT class membership but does not fully determine the latent classification. In other words, PCICT classes are not simply equivalent to categories of internet use frequency, but also reflect differences in how older adults perceive the convenience of ICT across multiple life domains.

Table S7. Distribution of internet use frequency across PCICT classes.

| PCICT class | Non-user, n (%) | Non-daily use, n (%) | Daily use, n (%) | Total, n |
| --- | --- | --- | --- | --- |
| All-domains Beneficiaries | 339 (15.72) | 122 (5.66) | 1,695 (78.62) | 2,156 |
| Foundational-domains Beneficiaries | 325 (17.22) | 333 (17.65) | 1,229 (65.13) | 1,887 |
| Extended-domains Frustrated | 566 (44.71) | 202 (15.96) | 498 (39.34) | 1,266 |
| Compound Experience | 2,154 (68.47) | 332 (10.55) | 660 (20.98) | 3,146 |
| Unaffected | 1,309 (89.35) | 57 (3.89) | 99 (6.76) | 1,465 |
| Total | 4,693 (47.31) | 1,046 (10.54) | 4,181 (42.15) | 9,920 |

*Note.* Percentages are row percentages for each PCICT class.

To further examine whether the associations between PCICT classes and health outcomes were mainly attributable to differences in internet use frequency, we re-estimated the regression models by additionally adjusting for internet use frequency. The results are presented in Table S8. After internet use frequency was included in the models, some coefficients were attenuated, but the overall pattern of the results remained unchanged. In addition, compared with non-users, daily internet users reported better self-rated health, fewer depressive symptoms, higher life satisfaction, and better social adaptation. Non-daily users reported better self-rated health and social adaptation, but more depressive symptoms, while their life satisfaction did not differ significantly from that of non-users.

These findings suggest that internet use frequency is itself associated with several health outcomes and may account for part of the observed differences across PCICT classes. However, the associations between PCICT classes and health outcomes remained generally consistent after adjustment for internet use frequency, indicating that these differences cannot be fully explained by internet use frequency. In summary, the main findings of associations between PCICT classes and health outcomes remained robust after accounting for internet use frequency.

Table S8. Associations between PCICT classes and health outcomes after adjustment for internet use frequency.

| VARIABLES | SRH | DS | LS | SA |
| --- | --- | --- | --- | --- |
| PCICT (Ref. Unaffected) |  |  |  |  |
| All-domains Beneficiaries | 0.167^***^ | -0.865^***^ | 0.160^***^ | 1.600^***^ |
|  | (0.029) | (0.121) | (0.028) | (0.154) |
| Foundational-domains Beneficiaries | 0.109^***^ | -0.389^**^ | 0.097^***^ | 0.393^**^ |
|  | (0.029) | (0.121) | (0.028) | (0.152) |
| Extended-domains Frustrated | 0.013 | 0.945^***^ | 0.117^***^ | 0.539^***^ |
|  | (0.030) | (0.123) | (0.029) | (0.159) |
| Compound Experience | 0.020 | -0.044 | 0.101^***^ | 1.129^***^ |
|  | (0.025) | (0.100) | (0.024) | (0.132) |
| Internet use frequency (Ref. non-users) |  |  |  |  |
| Non-daily use | 0.059^*^ | 0.385^***^ | 0.036 | 0.538^***^ |
|  | (0.027) | (0.113) | (0.026) | (0.141) |
| Daily use | 0.155^***^ | -0.637^***^ | 0.141^***^ | 1.103^***^ |
|  | (0.021) | (0.092) | (0.021) | (0.114) |
| Control variables | YES | YES | YES | YES |
| Constant | 4.162^***^ | 14.963^***^ | 3.721^***^ | 23.363^***^ |
|  | (0.108) | (0.444) | (0.105) | (0.571) |
| Observations | 9,920 | 9,920 | 9,920 | 9,920 |
| R^2^ | 0.207 | 0.193 | 0.152 | 0.179 |
| F | 55.95 | 113.0 | 38.56 | 46.75 |

*Note.* Standard errors in parentheses, ^***^ *p*<0.001, ^**^ *p*<0.01, ^*^ *p*<0.05, SRH = Self-rated health, DS = Depressive symptoms, LS = Life satisfaction, SA = Social adaptation.

We further repeated the indirect association analyses after additionally adjusting for internet use frequency. The results are presented in Table S9. After adjustment, the overall pattern of the indirect associations through ATA remained consistent with the main analyses. Compared with the Unaffected class, the All-domains Beneficiaries, Foundational-domains Beneficiaries, and Extended-domains Frustrated classes remained indirectly associated with better self-rated health, fewer depressive symptoms, higher life satisfaction, and better social adaptation through ATA. In contrast, the Compound Experience class showed indirect associations in the opposite direction across the four health outcomes. All of these indirect associations remained statistically significant after adjustment for internet use frequency. These results indicate that the indirect associations between PCICT classes and health outcomes through ATA cannot be fully explained by differences in internet use frequency, further supporting the robustness of the main findings. In summary, the indirect associations between PCICT classes and health outcomes remained robust after accounting for internet use frequency.

Table S9. Indirect associations between PCICT classes and health outcomes through ATA after adjustment for internet use frequency.

| Category | Total  effect | Direct  effect | Indirect  effect | Boot SE | LLCI | ULCI |
| --- | --- | --- | --- | --- | --- | --- |
| **PCICT - ATA- SRH** |  |  |  |  |  |  |
| All-domains Beneficiaries | 0.199^***^ | 0.167^***^ | 0.031^***^ | 0.005 | 0.022 | 0.040 |
| Foundational-domains Beneficiaries | 0.129^***^ | 0.109^***^ | 0.020^***^ | 0.004 | 0.013 | 0.027 |
| Extended-domains Frustrated | 0.030 | 0.013 | 0.016^***^ | 0.003 | 0.010 | 0.022 |
| Compound Experience | 0.009 | 0.020 | -0.011^**^ | 0.003 | -0.017 | -0.006 |
| **PCICT - ATA- DS** |  |  |  |  |  |  |
| All-domains Beneficiaries | -0.964^***^ | -0.865^***^ | -0.099^***^ | 0.016 | -0.131 | -0.067 |
| Foundational-domains Beneficiaries | -0.452^***^ | -0.389^**^ | -0.063^***^ | 0.012 | -0.087 | -0.039 |
| Extended-domains Frustrated | 0.905^***^ | 0.945^***^ | -0.040^***^ | 0.010 | -0.060 | -0.020 |
| Compound Experience | -0.009 | -0.044 | 0.035^***^ | 0.009 | 0.018 | 0.053 |
| **PCICT - ATA- LS** |  |  |  |  |  |  |
| All-domains Beneficiaries | 0.177^***^ | 0.160^***^ | 0.017^***^ | 0.004 | 0.009 | 0.025 |
| Foundational-domains Beneficiaries | 0.108^***^ | 0.097^***^ | 0.011^***^ | 0.003 | 0.005 | 0.016 |
| Extended-domains Frustrated | 0.126^***^ | 0.117^***^ | 0.009^***^ | 0.002 | 0.004 | 0.014 |
| Compound Experience | 0.094^**^ | 0.101^**^ | -0.007^**^ | 0.002 | -0.010 | -0.003 |
| **PCICT - ATA- SA** |  |  |  |  |  |  |
| All-domains Beneficiaries | 1.835^***^ | 1.600^***^ | 0.235^***^ | 0.029 | 0.178 | 0.292 |
| Foundational-domains Beneficiaries | 0.542^***^ | 0.393^**^ | 0.149^***^ | 0.024 | 0.101 | 0.196 |
| Extended-domains Frustrated | 0.626^***^ | 0.539^**^ | 0.087^***^ | 0.020 | 0.047 | 0.126 |
| Compound Experience | 1.053^***^ | 1.129^***^ | -0.076^***^ | 0.017 | -0.109 | -0.043 |

*Note.* Reference group= Unaffected; PCICT = Perceived convenience of information and communication technology, ATA = Attitudes toward aging, SRH = Self-rated health, DS = Depressive symptoms, LS = Life satisfaction, SA = Social adaptation; ^*^*p* < 0.05, ^**^*p* < 0.01, ^***^*p* < 0.001. Boot SE = bootstrap standard error; LLCI = lower limit of the 95% confidence interval; ULCI = upper limit of the 95% confidence interval.

References:

Austin, P. C. (2009). Balance diagnostics for comparing the distribution of baseline covariates between treatment groups in propensity‐score matched samples. *Statistics in Medicine*, *28*(25), 3083–3107. https://doi.org/10.1002/sim.3697

Kruskal, W. H., & Wallis, W. A. (1952). Use of Ranks in One-Criterion Variance Analysis. *Journal of the American Statistical Association*, *47*(260), 583–621. https://doi.org/10.1080/01621459.1952.10483441
